# Dentate nNOS accounts for stress‐induced 5‐HT_1A_ receptor deficiency: Implication in anxiety behaviors

**DOI:** 10.1111/cns.13269

**Published:** 2019-12-21

**Authors:** Li‐Juan Zhu, Chu Xu, Jie Ren, Lei Chang, Xian‐Hui Zhu, Nan Sun, Guo‐liang Meng, Meng‐Ying Liu, Jing Zhang, Yuan‐Yuan Li, Yu‐Lin Tang, Qi‐Gang Zhou

**Affiliations:** ^1^ Key Laboratory of Developmental Genes and Human Diseases MOE Department of Histology and Embryology School of Medicine Southeast University Nanjing China; ^2^ Department of Pharmacology School of Pharmacy Nanjing Medical University Nanjing China; ^3^ Institute of Neuroscience Soochow University Suzhou China; ^4^ Department of Clinical Pharmcay Sir Run Run Hospital Nanjing Medical University Nanjing China; ^5^ Department of Pharmacology School of Pharmacy Nantong University Nantong China; ^6^ Department of Pharmacy The Affiliated Drum Tower Hospital of Nanjing University Medical School Nanjing China

**Keywords:** 5‐HT_1A_ receptor, anxiety, chronic stress, glucocorticoids, neuronal nitric oxide synthase

## Abstract

**Background:**

Anxiety is a common disorder with high social burden worldwide. Dysfunction of serotonin‐_1A_ receptor (5‐HT_1A_ receptor) in the dentate gyrus (DG) of the hippocampus has been predominantly implicated in the anxiety behavior. However, the molecular mechanism underlying the deficiency of postsynaptic 5‐HT_1A_ receptor in regulating anxiety behavior remains unclear.

**Methods:**

Using pharmacological and genetic methods, we investigated the role of detate nNOS in 5‐HT_1A_ receptor decline and anxiety behavior induced by chronic mild stress (CMS) in mice.

**Results:**

Here we showed that local elevation of glucocorticoids in the DG accounted for chronic stress‐induced anxiety behavior. Neuronal nitric oxide synthase (nNOS) mediated chronic stress‐induced downregulation of 5‐HT_1A_ receptor in the DG through peroxynitrite anion (ONOO•) pathway but not cyclic guanosine monophosphate (cGMP) pathway. By using pharmacological tool drugs and nNOS knockout mice, we found that nNOS in the DG played a key role in chronic stress‐induced anxiety behavior.

**Conclusions:**

These findings uncovered an important role of nNOS‐5‐HT_1A_ receptor pathway in the DG of the hippocampus in chronic stress‐induced anxiety. Accordingly, we developed a “dentate nNOS‐5‐HT_1A_ receptor closed‐loop” theory (stress‐glucocorticoids‐nNOS‐Nitric oxide‐ONOO•‐5‐HT_1A_ receptor ‐nNOS) of stress‐related anxiety.

## 1 INTRODUCTION

Anxiety disorders are among the most disabling of all medical disorders.^1^ It has been well demonstrated that serotonin (5‐HT) neurotransmission plays important roles in modulation of numerous diseases.^2^, ^3^ These functions are mediated by at least 15 well identified different 5‐HT receptors.^4^, ^5^, ^6^ Among these receptors, 5‐HT_1A_ receptor, in particular, is proved to be implicated in the etiology of anxiety disorders.^7^ Several lines of evidence supported that 5‐HT_1A_ receptor level in the hippocampus of patients with anxiety disorders is in deficit and that partial 5‐HT_1A_ receptor agonists are anxiolytics.^8^, ^9^, ^10^, ^11^ Moreover, 5‐HT_1A_ receptor knockout mice represent a genetic animal model of anxiety.^7^, ^12^

Reportedly, stress is closely correlated with depression and anxiety.^13^ Chronic stress increases corticosterone (CORT, glucocorticoids in rodents), decreases 5‐HT_1A_ receptor in the dentate gyrus (DG), and gives rise to anxiety‐related behaviors.^14^, ^15^ Hence, glucocorticoids may be the predominant substance that mediates the impact of stress on the hippocampus in the development of anxiety. However, the molecular mechanisms underlying stress and glucocorticoids‐induced decrease in expression of hippocampal 5‐HT_1A_ receptor are still unclear.

Neuronal nitric oxide synthase (nNOS) is enriched in the hippocampus.^16^ Our previous study revealed that chronic stress upregulates the level and activity of hippocampal nNOS and thus contributes to stress‐induced depressive behaviors.^17^ There are some reports that nNOS inhibitors produce anxiolytic‐like effects, and 5‐HT system is dysfunctional in nNOS knockout mice.^18^, ^19^, ^20^ However, it remains unknown whether nNOS‐NO pathway contributes to 5‐HT_1A_ receptor deficiency in the development of anxiety. In physiological condition, NO functions through the soluble guanylate cyclase/cyclic guanosine monophosphate/protein kinase G (sGC/cGMP/PKG) pathway.^17^, ^21^ Besides, excessive NO, synthesized by increased nNOS, may react with O2·and thereby form peroxynitrite anion (ONOO•). Both NO itself and ONOO• can regulate a variety of molecular expression and function.^17^, ^21^, ^22^, ^23^ Importantly, it was found that sodium nitroprusside (SNP), a type of NO donors, causes anxiogenic effect at high doses but exerts anxiolytic property at low doses, implicating ONOO• in the induction of anxiety behaviors.^24^, ^25^, ^26^ Here, using genetic and pharmacological methods, we investigated the link among glucocorticoids, nNOS, and 5‐HT_1A_ receptor. We report that nNOS‐NO‐ONOO• pathway accounts for the high level of glucocorticoids‐induced decline in 5‐HT_1A_ receptor in the DG of the hippocampus and probably contributes to anxiety behavior, uncovering a novel molecular mechanism for anxiety pathogenesis.

## 2 MATERIALS AND METHODS

### 2.1 Animals

Young adult (6‐ to 7‐week‐old) male homozygous nNOS‐deficient mice (B6.129S4‐Nos1^tm1Plh^/J, KO, stock number: 002986) and their wild‐type controls (C57BL/6J, WT, stock number: 000664) (both from The Jackson Laboratory; maintained at Model Animal Research Center of Nanjing University, Nanjing, China), young adult (6‐ to 8‐week‐old) ICR mice were used for behavioral analysis. Littermates were used in a same individual experiment in this study. Animals were housed in an air‐conditioned room (20 ± 2°C), with food and water ad libitum, except when specified otherwise. Five mice were housed in each cage with 12 hours light/dark cycle. All procedures involving the use of animals were approved by the Institutional Animal Care and Use Committee of Nanjing Medical University (protocol number: IACUC‐1704010). Every effort was made to minimize the number of animals used and their suffering.

### 2.2 Drugs

For generating consistent elevated plasma level of CORT in mice, CORT (20 mg/kg/d) were injected subcutaneously. We selected the dosage due to that a low dose of CORT treatment induced anxiety‐like phenotype in mice.^27^ To block the synthesis of CORT continuously, we treated mice subcutaneously with metyrapone (100 mg/kg/d), the dosage of which was proved blocking the synthesis of CORT.^17^ To know the systemic effect of 7‐nitroindazole (7‐NI), 7‐NI was intraperitoneally injected at a dosage of 30 mg/kg/d which produced antidepressant‐like effect in mice.^28^ To study the effect of drugs in local sites, stereotaxic injection was used to deliver 10 μM corticosterone (CORT; 2 μL; Sigma‐Aldrich), 1 mM 5‐amino‐3‐(4‐morpholinyl)‐1,2,3‐oxadiazolium chloride (SIN‐1; 2μl; Sigma‐Aldrich), 10 μM 7‐NI (2 μl; Sigma‐Aldrich), or 20 μM water‐soluble carboxy‐2‐phenyl‐4,4,5,5‐tetramethylimidazolineoxyl‐1‐oxyl‐3‐oxide (cPTIO; 2 μL; Sigma‐Aldrich) into the bilateral DGs of the hippocampi. The designated drug solution (vehicle) in 2 μL of volume was microinjected (0.2 μL/min) into control mice. To avoid backflow of injected chemicals, the needle was kept in the injection site for 5 minutes before withdrawing of the needle. Diethylenetriamine NONOate (DETA/NONOate) and 1H‐[1,2,4]Oxadiazolo[4,3‐a]quinoxalin‐1‐one (ODQ) and spironolactone were purchased from Tocris Bioscience. For stereotaxic surgery, adult mice were anesthetized with 0.07 mL of a mixture of ketamine (90.9 mg/mL) and xylazine (9.1 mg/mL).

### 2.3 Chronic unpredicted mild stress

The procedure of chronic unpredictable mild stress (CUMS) was designed as described previously.^28^ Briefly, the CUMS protocol consists of the sequential application of a variety of mild stressors, including restraint in tubes, forced swimming in cold water, water and/or food deprivation, and pairing with another stressed animal in wet sawdust, reversal of the light/dark cycle, housing in wet sawdust, housing in constant illumination or darkness each for a period ranging from 10 minutes to 24 hours, in a schedule that lasts for 3 weeks. The schedule of stressors in the protocol is unpredicted to mice.

### 2.4 Behavioral measurements

For the open‐field test (OFT), the test apparatus was constructed of a plastic plate (72 × 72 cm). The under plate was into 256 squares by lines drawn on the plate. The plate was surrounded by a 35.18‐cm‐high plastic wall. The 64 squares in the center of the test arena were referred to as the central squares (36 × 36 cm). Each mouse was placed on a corner square of the arena, facing the corner, and allowed to freely explore the open field for 5 minutes per trial, recorded by Motor‐Monitor System SF16R. In the system, the squares are recognized and analyzed automatically. No stressor was applied to the animals for at least 12 hours before the test. After each trial, the plate was cleaned with 70% ethyl alcohol (EtOH). Mobility was scored when an animal crossed a sector border with both its hindlimbs. Parameters assessed were the number of square crossings (horizontal) and the times of standing (vertical) during the 5 minutes test. The value of parameters of each tested mouse was calculated by the Motor‐Monitor System SF16R automatically after tests. All the mice were measured about 24 hours after last injection of drugs. The experimental environment was evenly illuminated with white light (25 lux). Animals were group housed in a 12‐hour dark, 12‐hour light cycle and tests were performed during the light phase. Experimenter was blind as to the genotype of the mice in the behavior experiments.

### 2.5 Corticosterone level measurement

For measurement of CORT level, all mice were decapitated between 9:00 and 11:00 AM. Blood from angulus oculi vessels was collected in heparinized tubes, and CORT in plasma was measured with a CORT ELISA Kit according to the instructions of the manufacturer (Cayman Chemical Company). The detection limit of this kit is approximately 40 pg/mL. Mice were euthanized for plasma collection at least 15 hours after the end of behavior test.^29^

### 2.6 Cell culture

Primary hippocampal neurons were cultured as reported previously with minor modifications. In brief, the hippocampi of embryonic day 18 mice were freshly isolated and enzymatically dissociated in calcium‐ and magnesium‐free HBSS containing 0.125% trypsin at 37°C for 10 min. Then, the digestion was terminated with DMEM/F12 (1:1) containing 10% FBS. Dissociated cells were centrifuged, resuspended in Neurobasal medium (Invitrogen) containing 2% B27 supplement, 0.5 mM L‐glutamine, 5 IU penicillin, and 5 μg/mL streptomycin, and plated on 10 μg/mL polyornithine‐coated dishes (diameter, 3.5 cm) at 5 × 10^4^ cells/cm^2^. Half of the medium was replaced with fresh Neurobasal/B27 medium every 2‐3 days.

### 2.7 Western blot analysis

Western bolt analysis of samples from cultured hippocampal neurons and hippocampal tissues of animals was performed as described previously.^17^ In this study, we dissected the DG from the hippocampus for Western blot. The primary antibodies were as follows: rabbit anti‐nNOS (1:1000; Millipore Bioscience Research Reagents), mouse anti‐nitrotyrosine (1:3000; Millipore Bioscience Research Reagents), and rabbit anti‐5‐HT_1A_ receptor (1:500; Santa Cruz Biotechnology). Appropriate horseradish peroxidase‐linked secondary antibodies were used for detection by enhanced chemilumi‐nescence (Pierce; Thermo Fisher Scientific). When we prepared the samples, all the hippocampus was checked to find the needle track of infusion. Only the samples with obvious needle track of Alzet osmotic minipumps or infusion needles were remained for further measurement.

### 2.8 RNA extraction and reverse transcription‐PCR

Total RNA was extracted from the DG of the hippocampus using TRIzol reagent according to the manufacture's instructions (Sigma‐Aldrich). The primers for 5‐HT_1A_ receptor and GAPDH were as follows: for 5‐HT_1A_ receptor: Forward 5′‐GACTGCCACCCTCTGCCCTATATC‐3′ and Reverse 5′‐TCAGCAAGGCAAACAATTCCAG‐3′; For GAPDH: Forward 5′‐CAAGGTCATCCATGACAACTTTG‐3′ and Reverse 5′‐GTCCACCACCCTGTTGCTGTAG‐3′. PCR conditions were 30 cycles of denaturation at 94°C for 45 seconds, annealing at 55°C for 45 seconds, and extension at 72°C for 45 seconds. PCR products were separated by electrophoresis through 1.5% agarose gel containing 0.5% μg/mL ethidium bromide and imaged using a BioDoc‐IT imaging system (Bio‐Rad); band intensities were determined using GS‐710 calibrated imaging Densitometer (Bio‐Rad). The mRNA for GAPDH was included in the PCR mixture as a standard.

### 2.9 Microinjection

Adult mice were anesthetized with 0.07 mL of a mixture of ketamine (90.9 mg/mL) and xylazine (9.1 mg/mL) and placed in a stereotaxic apparatus (Stoelting). The drug solutions in 2 μL volume were microinjected into the DGs (0.2 μL/min) at coordinates 2.3 mm posterior to bregma, 1.3 mm lateral to the midline, and 2.0 mm below dura. Osmotic pump was used for continuous delivery of drug. For the osmotic pump implantation, two 7d or 28d Alzet osmotic minipumps (DURECT Corporation) containing cPTIO solution were placed subcutaneously in the back of mice, and two brain infusion cannulas connected to the pump were positioned at the following coordinates: 2.3 mm posterior to bregma, 1.3 mm lateral to the midline, and 2.0 mm below dura. The infusion rate of the osmotic pump was 0.25 μL/h. We anesthetized mice with a mixture of ketamine (90.9 mg/mL) and xylazine (9.1 mg/mL) and removed the osmotic pumps 1 day before behavioral tests.

### 2.10 Statistical analysis

Statistical analyses were performed using commercial software (GraphPad Prism; GraphPad Software Inc) Data are presented as Means ± SEM. To compare 3 or more independent groups passed normality test, evaluated by Shapiro‐Wilk normality test, were analyzed by one‐way ANOVA or two‐way ANOVA analysis. Comparisons among multiple groups were made by Bonferroni's post hoc test. To compare 3 or more independent groups which did not pass normality test, Kruskal‐Wallis tests with Dunn's multiple comparison corrections were used. To compare the effect of two factors with respect to abnormal distribution, we used two‐way ANOVA with Bonferroni's multiple comparison corrections. Comparisons between two groups were made with two‐tail Student's *t* test. To compare two independent groups with respect to abnormal distribution, Mann‐Whitney *U* tests were used. For all results, differences were considered significant when *P* < .05.

## 3 RESULTS

### 3.1 Elevated glucocorticoids in the hippocampus induce anxiety behaviors

**Figure 1:**
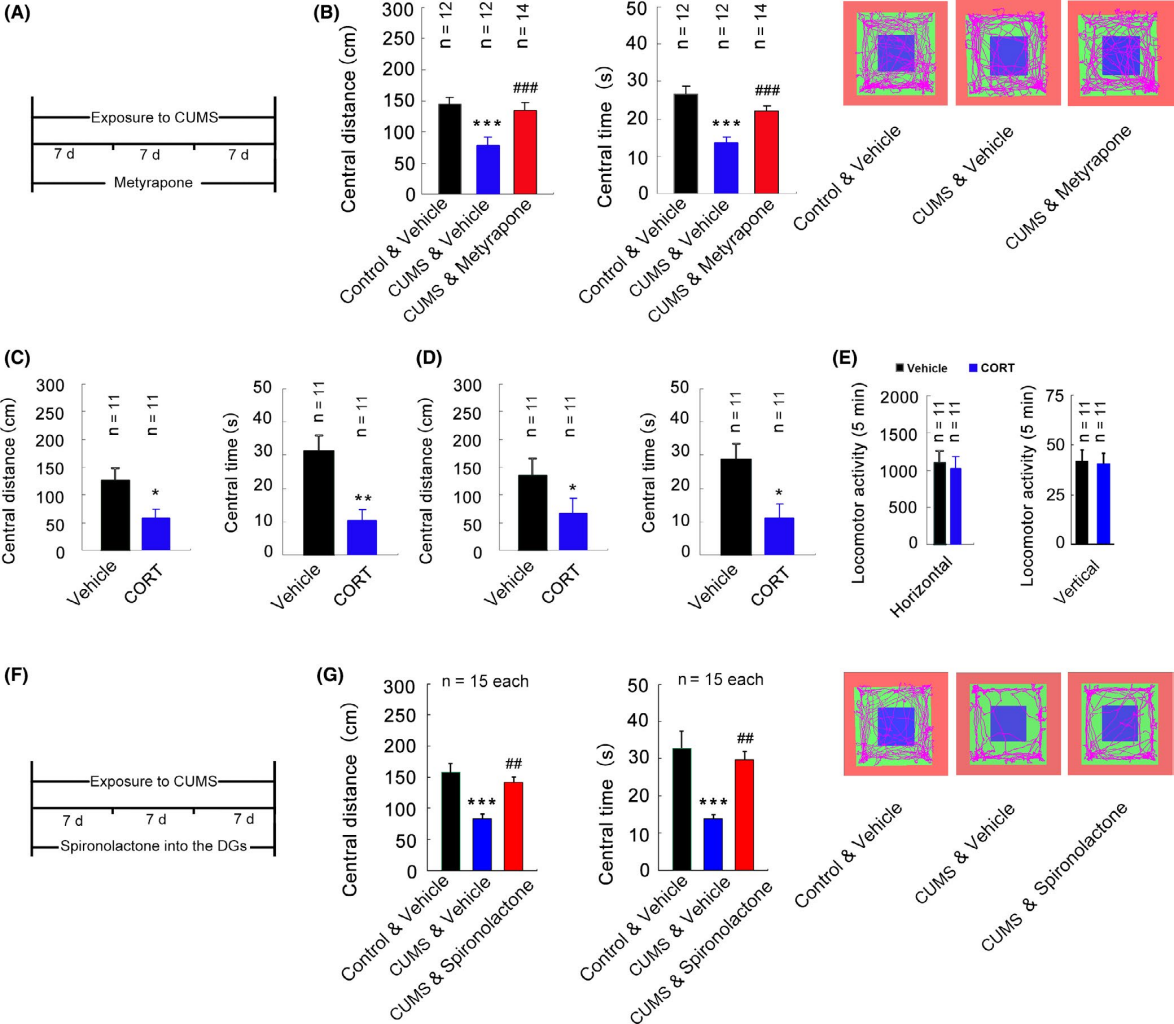
Elevated level of glucocorticoids in the DG causes anxiety behavior. (A) Schedule designed. Mice were administrated with or without metyrapone (100 mg/kg/d, s.c., 1 time/d, 21 d) during 21 d of exposure to CUMS. (B) Central distance (left) and central time (middle) spent by mice in the OFT after 21‐d CUMS with or without metyrapone administration (100 mg/kg, s.c., 1 time/d, 21 d). Representative tracks of mice in each group in the OFT are shown on the right. (C) Central distance (left) and central time (right) spent by mice in the OFT after 21 d CORT treatment (20 mg/kg, s.c. 1 time/d). (D) Central distance (left) and central time (right) spent by mice in the OFT 21 days after infusion of CORT (10 μM, 2 μL) into the DG. (E) The locomotor activity of mice in the OFT 21 days after infusion of CORT into the DG. Parameters assessed were the number of square crossings (horizontal) and the times of standing (vertical). (F) Schedule designed. Chronic continuouse delivery of spironolactone (50 μM, 0.25 μL/h) into the DGs using osmotic minipumps during 21 d of exposure to CUMS. (G) Central distance (left) and central time (middle) spent by mice in the OFT. Representative tracks of mice in each group in the OFT are shown on the right. Mean ± SEM, *P < .05, **P < .01, ***P < .001, compared with Vehicle, ^#^ *P* < .05, ^##^ *P* < .01, ^###^ *P* < .001, compared with CUMS + Vehicle in B and G, one‐way ANOVA for B and G. Student's *t* test for C, D, and E

CUMS paradigms, modeling the stressful life events, are applied as a typical model of depression and also induce anxiety behavior changes in rodents such as reduced time spent in open arm in elevated plus‐maze test, decreased time entering in central place in the OFT^30^, ^31^, ^32^. To know the role of glucocorticoids in stress‐induced anxiety‐like behaviors, we used metyrapone (at daily dose of 100 mg/kg, s.c. during 21‐day CUMS exposure, Figure 1A), a corticosteroids synthesis inhibitor,^17^ to block CORT synthesis and to test behavioral changes in the OFT. In consistent with our previous study, the mice, which underwent 21‐day CUMS, possessed a markedly a higher concentration of CORT in the plasma (*F*_2,14_ = 28.98, *P* < .001; Vehicle: 73.59 ± 21.90 ng/mL; CUMS + Vehicle: 236.40 ± 55.80 ng/mL; Control + Vehicle vs. CUMS + Vehicle: *P* < .001, n = 5‐6, one‐way ANOVA) and exhibited reduced central distance and central time in the OFT (Figure 1B, central distance: *F*_2,35_ = 36.90, *P* < .001, Control + Vehicle vs. CUMS + Vehicle: *P* < .05; central time: *F*_2,35_ = 35.24, *P* < .001, CUMS + Vehicle vs. Control + Vehicle vs. CUMS + Vehicle: *P* < .05), reflecting anxiety phenotype in mice. Indeed, metyrapone treatment blocked the increase in CUMS‐induced CORT synthesis (*F*_2,14_ = 28.98, *P* < .001; CUMS + Vehicle: 236.40 ± 55.80 ng/mL; CUMS + Metyrapone: 77.64 ± 30.09 ng/mL; CUMS + Vehicle vs. CUMS + Metyrapone: *P* < .001. n = 5‐6, one‐way ANOVA). Importantly, metyrapone normalized the central distance and central time spent by the mice exposed to CUMS in the OFT (Figure 1B, central distance: *F*_2,35_ = 36.90, *P* < .001, CUMS + Vehicle vs. CUMS + Metyrapone: *P* < .05; central time: *F*_2,35_ = 35.24, *P* < .001, CUMS + Vehicle vs. CUMS + Metyrapone: *P* < .05). Additionally, all treatments did not change the locomotor activity of mice (data not shown). Together, these results indicate that the elevated level of CORT in the body accounts for chronic stress exposure‐induced anxiety behavior.

To further investigate whether high level of glucocorticoids per se can directly induce anxiety‐like behavior, we treated mice with CORT (20 mg/kg, s.c., once per day) for 21 days and found that chronic administration with CORT caused a significant decrease in central distance and central time spent by the mice in the OFT (Figure 1C, Student's *t* test, central distance: *P* < .05; central time: *P* < .01). However, treatments did not change the locomotor activity of mice (data not shown). Next, to study whether local elevation of CORT in the DG induce anxiety‐like behavior, we microinjected CORT (10 μM, 2 μL) into the bilateral DGs of the hippocampi and measured the anxiety‐related behaviors 21 days later. As expected, CORT microinjection resulted in a significant decrease in central distance and central time spent by the mice in open‐field test (Figure 1D, Student's *t* test, central distance: *P* < .05; central time: *P* < .05) but did not change the locomotor activity (Figure 1E, Student's *t* test, Horizontal: *P* > .05; Vertical: *P* > .05). To know whether blocking the function of corticosteroids locally in bilateral DGs can affect the effect of stress on anxiety behavior, we infused the glucocorticoids receptor antagonist, spironolactone (50 μM, 0.25 μL/h), into the DGs using osmotic minipumps during CUMS. Local administration of spironolactone into the DGs reversed CUMS‐induced decreased central distance and central time spent by the mice exposed to CUMS in the OFT (Figure 1B, central distance: *F*_2,42_ = 12.73, *P* < .001, CUMS + Vehicle vs. CUMS + Spironolactone: *P* < .01; central time: *F*_2,42_ = 2.407, *P* < .001, CUMS + Vehicle vs. CUMS + Spironolactone: *P* < .01). Collectively, these data demonstrate that elevated glucocorticoids in the DG of the hippocampus play a critical role in the induction of stress‐related anxiety behavior.

### 3.2 Hippocampal nNOS mediates the glucocorticoids‐induced 5‐HT_1A_ receptor expression decline

**Figure 2:**
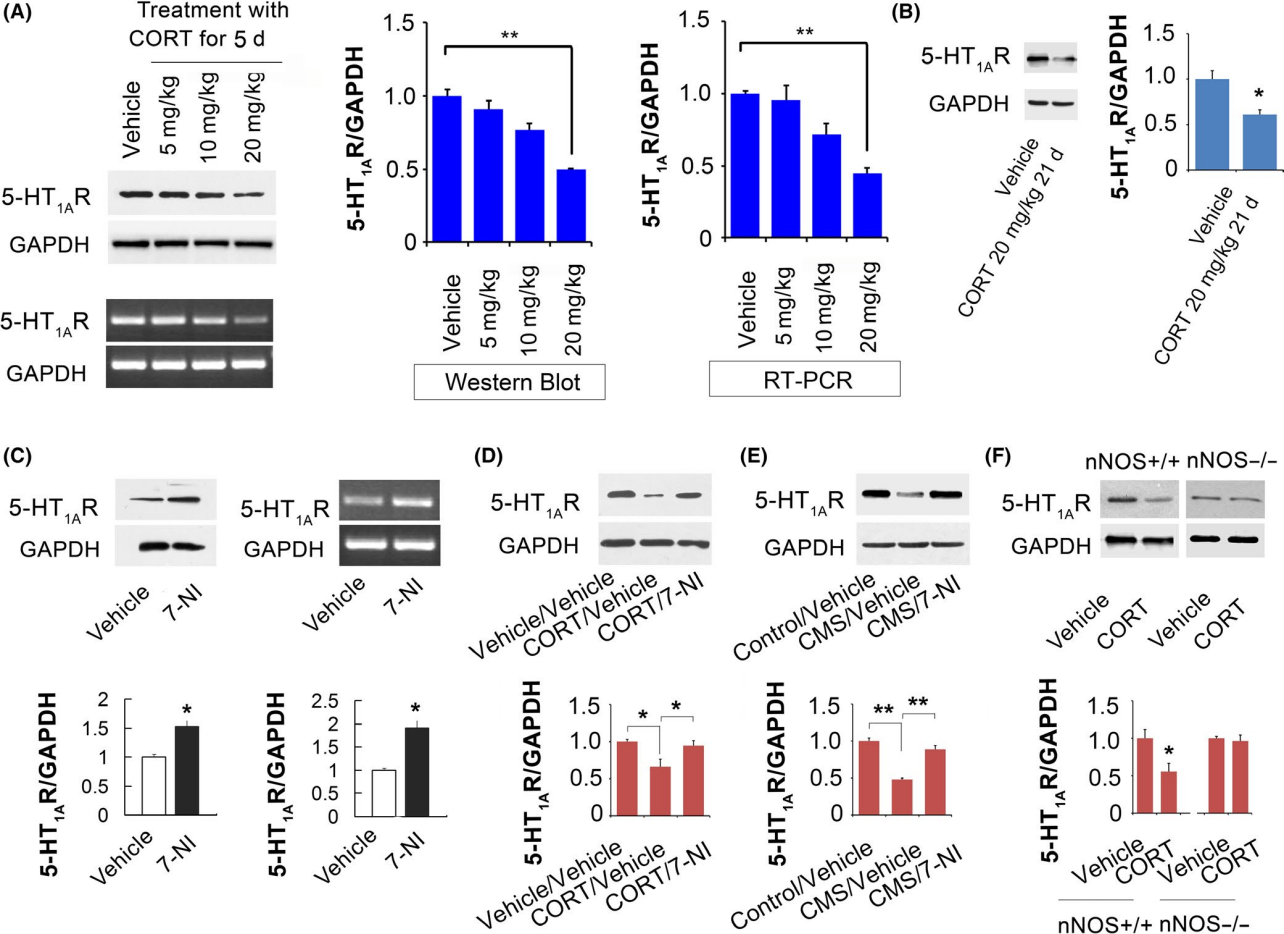
Dentate nNOS accounts for high concentration of glucocorticoids‐induced 5‐HT_1A_ receptor expression decline. (A) Representative protein (upper) and mRNA level (lower) of 5‐HT_1A_ receptor and GAPDH in the DG of mice treated with different concentration of CORT (5, 10, and 20 mg/kg, s.c., 1 time per day) and vehicle for 5 d. The corresponding bar graphs are on the right side. (B) Representative Western blot of 5‐HT_1A_ receptor and GAPDH in the DG of mice treated with high concentration of CORT (20 mg/kg, s.c., 1 time per day) and vehicle for 21 d. Bar graph shows the analysis. (C) Representative Western blot (left) and RT‐PCR (right) of 5‐HT_1A_ receptor and GAPDH in the DG of mice treated with 7‐NI (10 mg/kg, i.p., 1 time per day) and vehicle for 5 d. Bar graphs show the analysis. (D) Representative Western blot of 5‐HT_1A_ receptor and GAPDH in the DG of mice treated with CORT (20 mg/kg, s.c., 1 time per day) and vehicle for 5 d with or without 7‐NI infusion into the DG the day before the beginning of CORT administration. Bar graph shows the analysis. (E) Representative Western blot of 5‐HT_1A_ receptor and GAPDH in the DG of mice exposed to CUMS for 5 d with or without 7‐NI infusion into the DG the day before the beginning of CUMS. Bar graph shows the analysis. (F) Representative Western blot and bar graph analysis of 5‐HT_1A_ receptor expression in the hippocampus of nNOS−/− and nNOS+/+ (WT) mice treated with CORT (20 mg/kg, s.c., 1 time per day) and vehicle for 21 d. Mean ± SEM, *P < .05, **P < .01. One‐way ANOVA for A, D, and E. Two‐way ANOVA for F. Student's *t* test for B and C

Downregulation of 5‐HT_1A_ receptor in DG granule cells of the hippocampus is essential for induction of anxiety.^33^ By Western blot and RT‐PCR methods, we found that a high dose of CORT (20 mg/kg, s.c., 5 days) but not lower doses (10 and 5 mg/kg, s.c., 5 days) of CORT significantly reduced the protein and mRNA level of 5‐HT_1A_ receptor in the DG of the hippocampus compared with vehicle‐treatment group (Figure 2A, one‐way ANOVA, Western blot: *F*_3,8_ = 10.36, *P* < .01, n = 3; RT‐PCR: *F*_3,8_ = 11.55, *P* < .01, n = 3). Notably, persistent inhibition of 5‐HT_1A_ receptor protein expression in the DG was also observed after long‐term administration of CORT (20 mg/kg, s.c., 21 days, Figure 2B, Western blot: Student's *t* test, *P* < .05, n = 4).

To know whether nNOS is involved in the regulation of 5‐HT_1A_ receptor by stress and glucocorticoids, first, we treated mice with 7‐NI (30 mg/kg, i.p., 5 days), a selective nNOS activity inhibitor,^28^ and found that hippocampal nNOS negatively regulated both the protein and mRNA level of 5‐HT_1A_ receptor in the DG (Figure 2C, Student's *t* test, Western blot: *P* < .05, n = 3, RT‐PCR: *P* < .05, n = 3). Second, we selectively infused 7‐NI (10 μM, 2 μL) into the bilateral DGs of the hippocampi followed by high concentration of CORT administration (20 mg/kg, s.c., Figure 2D) or CUMS exposure for 5 days (Figure 2E). The results indicated that inhibition of nNOS catalytic function in the DG blocked the negative regulation of 5‐HT_1A_ receptor expression in the hippocampus by CORT (Figure 2D, one‐way ANOVA, *F*_2,6_ = 70.44, *P* < .001, n = 3) and CUMS (Figure 2E, one‐way ANOVA, *F*_2,6_ = 10.89, *P* < .05, n = 3). The precision of the coordinates used for local infusion into the DG was validated by microinjection of DiIC18(3) (DiI), a type of red color dye, into the DG (data not shown). Moreover, we found that CORT administration (20 mg/kg, s.c., 21 days) significantly reduced the hippocampal 5‐HT_1A_ receptor expression in wild‐type (WT) mice (Figure 2F, Student's *t* test, *P* < .05, n = 5) but not in nNOS−/− mice (Figure 2F, Student's *t* test, *P* > .05, n = 5). Together, nNOS plays a crucial role in the stress or glucocorticoids‐induced 5‐HT_1A_ receptor expression decline in the DG of the hippocampus.

### 3.3 nNOS downregulates 5‐HT_1A_ receptor expression via ONOO•

**Figure 3:**
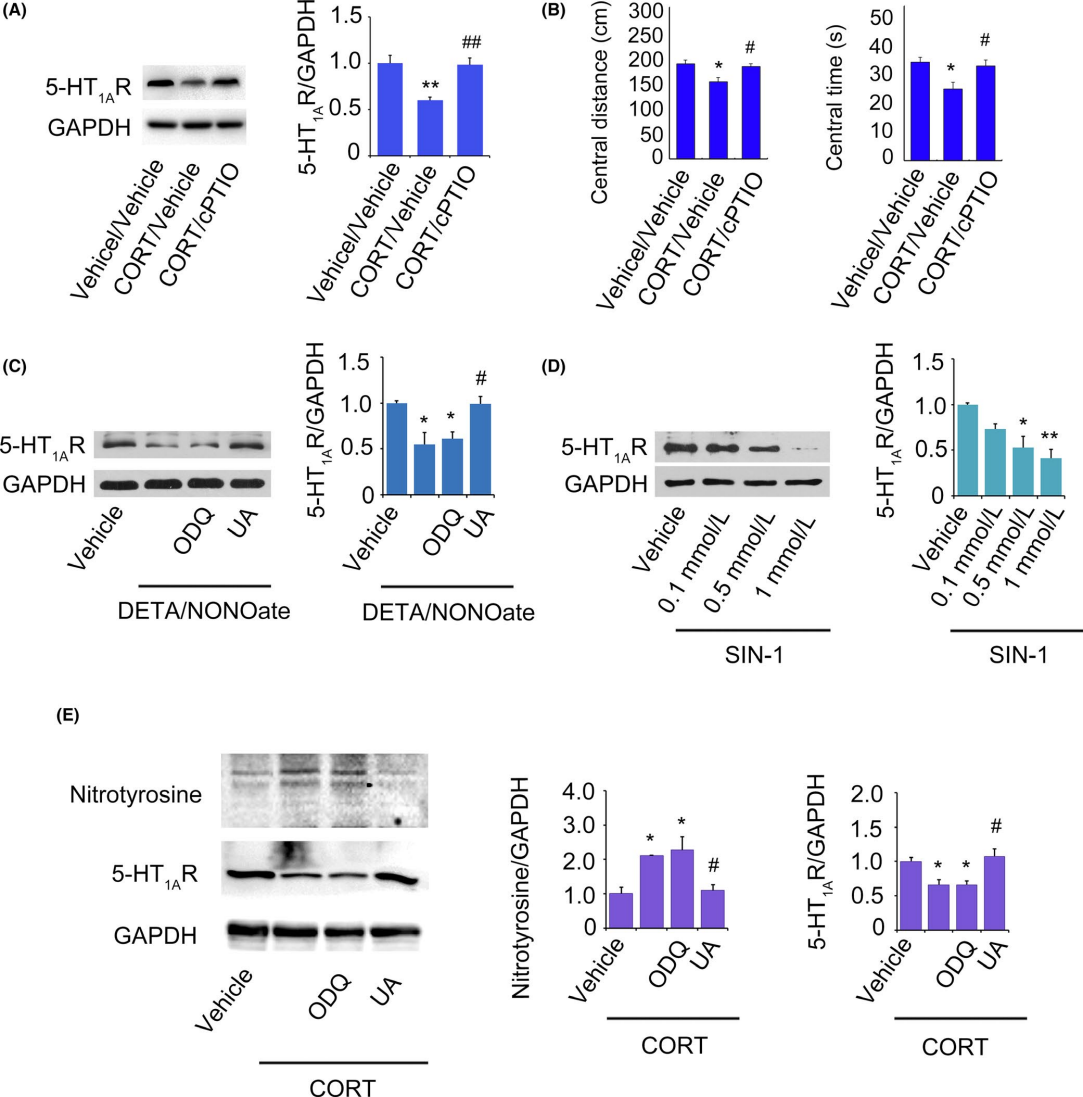
ONOO• pathway is implicated in the negative modulation of 5‐HT_1A_ receptor expression by nNOS. (A) Representative Western blot and bar graph analysis of 5‐HT_1A_ receptor in the hippocampus of mice administrated with CORT (20 mg/kg, s.c., 5 d, 1 time per day) and vehicle with or without cPTIO or vehicle infusion into the DGs. (B) Central distance (left) and central time (right) spent by mice in the open‐field test after CORT (20 mg/kg, s.c., 21 d, 1 time per day) and vehicle administration with or without cPTIO or vehicle infusion. (C) Representative Western blot of 5‐HT_1A_ receptor and GAPDH in cultured hippocampal neurons incubated with DETA/NONOate and vehicle together with or without ODQ or UA or vehicle. Measurement is from 4 different samples. (D) Representative Western blot of 5‐HT_1A_ receptor in cultured hippocampal neurons incubated with different concentration of SIN‐1 and vehicle. Measurement is from 3 different samples. (E) Representative Western blot of nitrotyrosine and 5‐HT_1A_ receptor in hippocampal neurons incubated with CORT and vehicle together with or without ODQ or UA or vehicle. Measurement is from 3 different samples. Mean ± SEM, *P < .05, **P < .01, compared with vehicle or vehicle/vehicle; ^#^ *P* < .05, ^##^ *P* < .01, compared with CORT/Vehicle in A and B, DETA/NONOate in C, CORT in E, one‐way ANOVA

NO mediates the main biological function of nNOS.^22^ To determine the role of NO in the regulation of 5‐HT_1A_ receptor by CORT, we cleared endogenous NO by cPTIO, a tool drug that directly extinguishes NO generated by NO synthase (NOS) without affecting NOS activity.^17^ The cPTIO was infused into the DGs via osmotic minipumps (Alzet, 20 μM, 0.25 μL/h) at day 1, followed by 5 days CORT administration (20 mg/kg, s.c., once per day). Western blots analysis showed that hippocampal 5‐HT_1A_ receptor expression remained unchanged after CORT exposure together with NO clearance compared with vehicle exposure, while CORT administration without NO clearance significantly reduced the expression of 5‐HT_1A_ receptor expression in the DG (Figure 3A, one‐way ANOVA, *F*_2,6_ = 9.34, *P* < .05, n = 3). Next, the cPTIO was infused into the DGs via osmotic minipumps at day 1 (20 μM, 0.25 μL/h) followed by CORT administration (20 mg/kg, s.c., once per day, 21 days). Interestingly, consistently, chronic CORT exposure (20 mg/kg, s.c., 1 time per day) did not induce anxiety behaviors after hippocampal NO clearance by cPTIO (Figure 3B, one‐way ANOVA, central distance: *F*_2,30_ = 7.60, *P* < .01; central time: *F*_2,30_ = 5.53, *P* < .01, n = 10‐13).

The sGC‐cGMP‐PKG pathway and reactive peroxynitrite anion (ONOO•) are the two major physiological signaling mechanisms of NO bioactivity.^21^ The question is how NO regulates 5‐HT_1A_ receptor expression? To know this, we incubated hippocampal neurons with high concentration of DETA/NONOate (100 μM), a NO donor, in combination with ODQ (10 μM), an inhibitor of the sGC‐cGMP‐PKG pathway, or uric acid (UA, 1.0 mM), a natural scavenger of ONOO•,^17^ at DIV14 for 24 hours. As shown in Figure 3C, DETA/NONOate markedly reduced the expression of 5‐HT_1A_ receptor, which was reversed by UA but not ODQ (one‐way ANOVA, *F*_3,12_ = 8.22, *P* < .01, n = 4, DETA/NONOate vs. DETA/NONOate & ODQ, *P* > .05; DETA/NONOate vs. DETA/NONOate & UA, *P* < .05), suggesting that the production of ONOO• accounts for the decrease in 5‐HT_1A_ receptor induced by DETA/NONOate. To further confirm the effect of ONOO• on 5‐HT_1A_ receptor, we applied SIN‐1 (0.1, 0.5, and 1 mM), an “NO donor” that generates NO and superoxide and then react to produce a large amount of ONOO• in cultured hippocampal neurons at DIV 14 for 24 hours. As expected, the expression of 5‐HT_1A_ receptor was diminished by SIN‐1 in a dose‐dependent manner (Figure 3D, one‐way ANOVA, *F*_3,8_ = 8.92, *P* < .01, n = 3). More importantly, the high concentration of CORT exposure (10 μM) led to a high level of nitrotyrosine and reduced the content of 5‐HT_1A_ receptor in the cultured hippocampal neurons at DIV 14 for 24 hours, which were reversed by UA but not ODQ incubation for 24 hours (Figure 3E, n = 3). More importantly, we infused DETA/NONOate (100 μM) together with ODQ (10 μM) or UA (1.0 mM) into the DG via minipumps, and 5 days later, we collected the DGs for protein level measurement. Consistent with the results of drugs incubated in cultured hippocampal neurons, DETA/NONOate significantly decreased the expression of 5‐HT_1A_ receptor in vivo, which also was reversed by UA but not ODQ (data not shown, one‐way ANOVA, *F*_3,8_ = 10.14, *P* < .01, n = 3, DETA/NONOate vs. DETA/NONOate & ODQ, *P* > .05; DETA/NONOate vs. DETA/NONOate & UA, *P* < .05), suggesting that ONOO•·but not sGC‐cGMP‐PKG pathway accounts for the decrease in 5‐HT_1A_ receptor expression induced by excess NO in the DG. Taken together, these results suggest that the production of excessive ONOO• caused by high level of glucocorticoids results in 5‐HT_1A_ receptor expression diminish in the DG.

### 3.4 Hippocampal nNOS mediates the glucocorticoids‐induced anxiety behaviors

**Figure 4:**
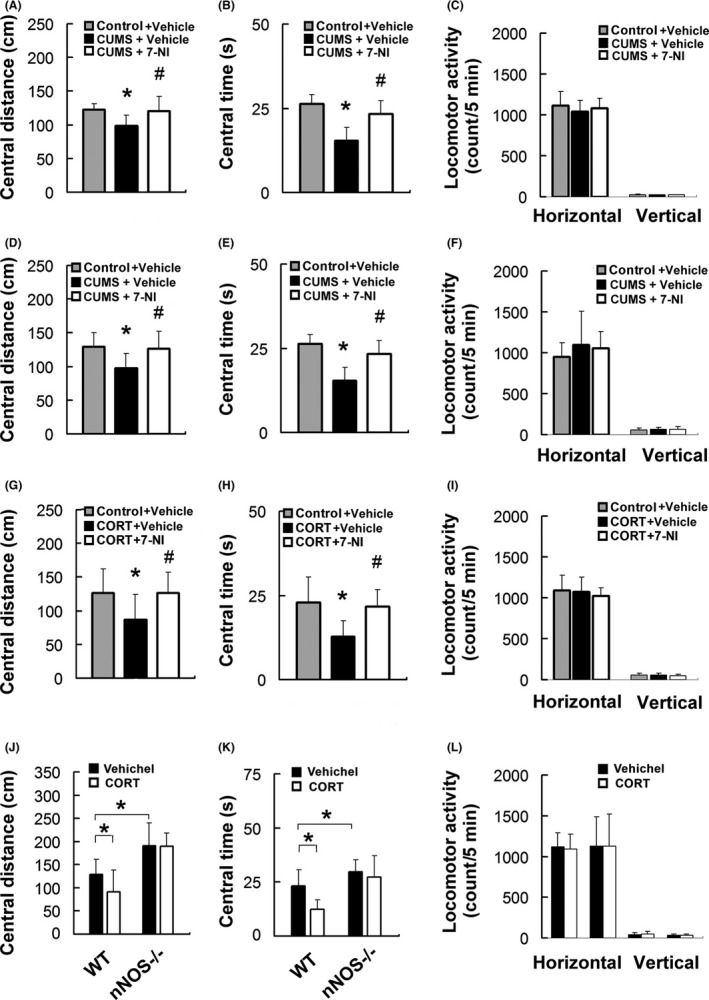
Dentate nNOS inhibition rescues glucocorticoids‐induced anxiety‐related behaviors. (A) Central distance of the mice exposed to CUMS for 21 d and the mice treated with 7‐NI accompanied with CUMS for 21 d in open‐field test. (B) Central time of the mice exposed to CUMS for 21 d and the mice treated with 7‐NI accompanied with CUMS for 21 d in open‐field test. (C) CUMS and 7‐NI have no effect on locomotor activity. (D) Central distance of the mice exposed to CUMS for 21 d and the mice treated with 7‐NI infusion into hippocampus followed by CUMS for 21 d in open‐field test. (E) Central time of the mice exposed to CUMS for 21 d and the mice treated with 7‐NI infusion into hippocampus followed by CUMS for 21 d. (F) CUMS and 7‐NI infused into hippocampus have no effect on locomotor activity. (G) Central distance of the mice with treatment of CORT for 21 d and the mice treated with 7‐NI infusion into hippocampus followed by CORT treatment for 21 d in open‐field test. (H) Central time of the mice with treatment of CORT for 21 d and the mice treated with 7‐NI infusion into hippocampus followed by CORT treatment for 21 d. (I) CORT and 7‐NI infused into hippocampus have no effect on locomotor activity. (J) Central distance of nNOS+/+ (WT) and nNOS−/− mice with treatment of CORT. (K) Central time of nNOS+/+ and nNOS−/− mice with treatment of CORT. (L) Locomotor activity of the nNOS−/− and nNOS+/+ mice with treatment of CORT. Parameters assessed were the number of square crossings (horizontal) and the times of standing (vertical). Mean ± SEM, *P < .05, compared with control or vehichel; #*P* < .05, compared with CUMS or CORT. One‐way ANOVA for A‐I, two‐way ANOVA for J‐L

To know the role of nNOS in chronic stress‐induced anxiety, we exposed adult mice to CUMS for 21 days with or without treatment with 7‐NI (30 mg/kg, i.p., 21 days). Systematic inhibition of nNOS activity prevented anxiety behavior change (Figure 4 A‐B, one‐way ANOVA, central distance: *F*_2,33_ = 7.45, *P* < .01; central time: *F*_2,33_ = 29.00, *P* < .001, n = 10‐12) after CUMS exposure without locomotor activity alteration as measured in the OPT (Figure 4C). Next, we infused 7‐NI (10 μM, 2 μL) into bilateral DGs of the hippocampi followed by 3 weeks CUMS exposure. Behavior measurement in the OPT showed that selective inhibition of nNOS located in the DG blocked the induction of anxiety behavior by CUMS (Figure 4 D‐E, one‐way ANOVA, central distance: *F*_2,32_ = 6.78, *P* < .01; central time: *F*_2,32_ = 7.74, *P* < .01, n = 11‐13). Meanwhile, the locomotor activity did not interrupt the anxiety behavior measurement (Figure 4F). To directly investigate whether the local nNOS in the DG account for glucocorticoids‐induced anxiety behavior, we infused 7‐NI (10 μM, 2 μL) into the DGs and administrated the mice with CORT (20 mg/kg, s.c.) for 21 days. Remarkably, nNOS activity inhibition in the DG blocked CORT‐induced anxiety behavior in the OFT (Figure 4G‐H, one‐way ANOVA, central distance: *F*_2,29_ = 4.47, *P* < .05; central time: *F*_2,29_ = 8.80, *P* < .01, n = 10‐11) without affecting the locomotors (Figure 4I). Consistently, 21 days CORT exposure (20 mg/kg, s.c.) caused anxiety behavior in WT mice but not in nNOS−/− mice (Figure 4J‐K, two‐way ANOVA, central distance: *F*_3,44_ = 14.69, *P* < .001, WT vehicle vs. WT CORT, *P* < .05, nNOS−/− vehicle vs. nNOS−/− CORT, *P* > .05; central time: *F*_3,44_ = 8.80, *P* < .01, WT vehicle vs. WT CORT, *P* < .05, nNOS−/− vehicle vs. nNOS−/− CORT, *P* > .05, n = 10‐13). Meanwhile, the locomotor activity did not interrupt the anxiety behavior measurement (Figure 4L). These data suggest that nNOS in the hippocampal DG mediates the chronic stress and glucocorticoids‐induced anxiety behavior change.

## 4 DISCUSSION

Extensive evidences demonstrate that 5‐HT_1A_ receptor deficiency is implicated in the pathogenesis of anxiety and that agonists of 5‐HT_1A_ receptor have anxiolytic effect in clinical use.^8^, ^11^, ^29^ However, it remains an important question unresolved that how 5‐HT_1A_ receptor become deficiency during the development of anxiety? Previous studies from our laboratory found that chronic stress and glucocorticoids exposure increased the expression of nNOS in the DG. Here, we report a causal link between the nNOS and the deficiency of the 5‐HT_1A_ receptor in the DG of the hippocampus after high dosage of glucocorticoids (corticosterone in rodent) exposure, which account for the generation of anxiety behavior after chronic stress. Interestingly, we found that ONOO• pathway but not cGMP pathway of NO was involved in the downregulation of 5‐HT_1A_ receptor expression.

The serotonergic system is a main pathway accounting for anxiety disease. Postsynaptic 5‐HT_1A_ receptor is expressed at a high density in the hippocampus.^34^ Decreased content of 5‐HT_1A_ receptor is found in the hippocampus of patients with anxiety and depression disorder.^11^ Re‐expression of 5‐HT_1A_ receptor primarily in the hippocampus and cortex is sufficient to rescue the anxiety phenotype of the 5‐HT_1A_ receptor knockout mice.^7^ The hippocampal CA1 and DG densely expresses 5‐HT_1A_ receptor.^35^ In the DG, 5‐HT_1A_ receptor majorly expresses on dentate granular cells.^35^, ^36^ Both transgenic and pharmacological evidences showed that 5‐HT1A receptor on dentate granular cells is engaged for depression and anxiety pathology, and antidepressant response.^29^, ^33^, ^37^ However, the molecular mechanism responsible for the key pathophysiological process, the dysfunction of 5‐HT_1A_ receptor on dentate granular cells, remains obscure. It was reported that glucocorticoids have a profound influence on the function of the hippocampus via regulating several lines of genes.^38^ Our previous study found that glucocorticoids modulate HPA activity through nNOS‐GR (glucocorticoid receptors) pathway.^17^ ONOO• produced by the diffusion‐controlled reaction of NO with superoxide O_2_^‐^ radical can regulate the expression of translational factors and proteins.^22^ Our data suggested that ONOO• mediated the repressive effect on 5‐HT_1A_ receptor expression after glucocorticoids action. Thus, it is possible that the enhanced concentration of NO in response to glucocorticoids exposure provides more NO diffused into granule cells in the DG to produce ONOO•. Consistently, it was reported that a high dose of SNP, a type of NO donor causes an anxiogenic‐like action in the elevated plus‐maze test.^24^ It has been demonstrated that 5‐HT_1A_ receptor on the hippocampal DG granule cells is crucial for anxiety‐related behavior.^33^ Thus, excessive ONOO• eliminates the content of 5‐HT_1A_ receptor in granule cells, which may finally induce anxiety phenotype. The OFT is a common measure of general locomotor activity levels and anxiety in rodent. Beside OFT, EPM and LDT are another two widely used behavior tests for assessment of anxiety. In this study, the anxiety behavior only assessed in OFT. Thus, more evidence for confirmation of the role of glucocorticoids‐nNOS‐Nitric oxide‐ONOO•‐5‐HT1A receptor pathway in stress‐related anxiety is needed.

Benzodiazepines, a type of typical anxiolytics, exert anxiolytic effects by regulating 5‐HT release in the ventral hippocampus.^39^ Recently, it is fully demonstrated that 5‐HT_1A_ receptor on mature dentate gyrus granule cells are critical for anxiety‐ and depression‐related behaviors in rodents.^33^ Stress potently modulates anxiety‐ and depression‐related behaviors.^13^, ^28^, ^40^ For instance, it has been demonstrated that foot shock stress as well as restraint stress can induce anxiety behavior in mice and rats.^41^, ^42^ Furthermore, 5‐HT_1A_ receptor expression decrease is strongly implicated in the pathology of anxiety and 5‐HT_1A_ receptor reactivation is critical for the anxiolytic effect of SSRIs.^43^, ^44^

In recent years, the function of adult hippocampal neurogenesis in mediating the effects of antidepressants has been realized.^8^, ^45^ Treatment of antidepressants increased hippocampal neurogenesis in human and rodents.^46^, ^47^ Disruption of hippocampal neurogenesis by X‐ray irradiation blocked the anxiolytic action of fluoxetine.^8^ Long‐term antidepressant treatment activated 5‐HT_1A_ receptor in the forebrain and 5‐HT_1A_ receptor activation in the hippocampus was required for the treatment of anxiety‐associated behaviors.^29^, ^48^ Recently, we discovered that hippocampal neurogenesis is crucial for 5‐HT_1A_ receptor in modulating anxiety behaviors.^37^, ^49^ Here, we found that a single intrahippocampal injection of 7‐NI into the DG counteracted the anxiety behavior of chronic stress or systemic corticosterone administration. Impaired hippocampal neurogenesis contributed to chronic stress and elevated glucocorticoids‐induced depression and anxiety behavior.^27^, ^28^ Moreover, nNOS knockout and 7‐NI significantly enhanced hippocampal neurogenesis.^28^ Therefore, the 5‐HT_1A_ receptor reactivation‐induced enhancement of hippocampal neurogenesis might contribute to the anxiety behavior rescue after a single infusion of 7‐NI.

**Figure 5:**
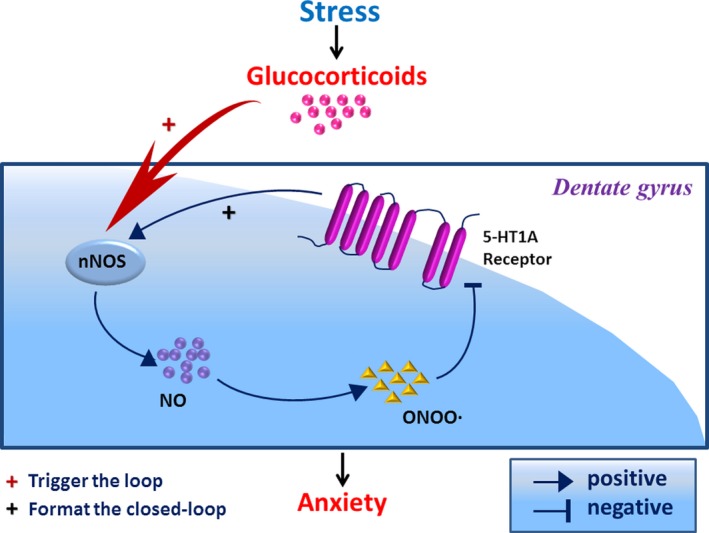
A proposed model of nNOS‐ONOO•‐5‐HT_1A_ receptor pathway in stress‐induced anxiety. High level of glucocorticoids‐induced dentate nNOS overexpression accounts for 5‐HT_1A_ receptor expression decline in the DG after stress. Our previous study demonstrated that hippocampal 5‐HT_1A_ receptor expression reduction results in nNOS overexpression. Accordingly, the reduction of hippocampal 5‐HT_1A_ receptor after stress, in turn, further increases the expression of nNOS, which is named as “nNOS‐5‐HT_1A_ receptor closed‐loop.” ONOO• mediates the downregulation of 5‐HT_1A_ receptor by nNOS. Overall, the glucocorticoids‐triggered “dentate nNOS‐5‐HT_1A_ receptor closed‐loop” plays a critical role in the pathology development of stress‐induced anxiety

In our previous study, we found that blockade of 5‐HT_1A_ receptor increased nNOS expression 29 (Figure 5, demonstrated previously part). Downregulation of hippocampal nNOS expression mediated the anxiolytic effects of fluoxetine and 5‐HT_1A_ receptor agonists.^29^ These findings suggest that nNOS works as a downstream molecule in the 5‐HT_1A_ receptor cascade, explaining the molecular basis of anxiolytic effects of selective serotonin reuptake inhibitor. However, the function of hippocampal nNOS in stress‐induced anxiety remains unclear. Here, we went one more step to understand the pathology of stress‐induced anxiety. We found that hippocampal nNOS accounted for chronic stress‐induced decrease in 5‐HT_1A_ receptor expression via ONOO• pathway (Figure 5). Previously, we demonstrated that high concentration of glucocorticoids after stress upregulated nNOS via mineralocorticoid receptor (MR) in the DG of the hippocampus.^17^ Altogether, these evidences support a novel hypothesis of anxiety that chronic stress‐induced nNOS overexpression reduce the expression of 5‐HT_1A_ receptor in the DG, causing a higher level of nNOS, which in turn lead to more severe deficiency of 5‐HT_1A_ receptor in the DG. We, thus, name this undesirable interaction between nNOS and 5‐HT_1A_ receptor as “nNOS‐5‐HT_1A_ receptor loop” (stress‐glucocorticoids‐nNOS‐Nitric oxides‐ONOO•‐5‐HT_1A_ receptor ‐nNOS), which functions importantly in the pathology of stress‐associated anxiety behavior (Figure 5). We also illustrated that ONOO• plays an important role in the “nNOS‐5‐HT_1A_ receptor loop” under chronic stress state. Additionally, we clarified the mechanism by which 5‐HT_1A_ receptor modulated nNOS.^29^ The coordinate used for microinjection in this study specially target the DG of the ventral hippocampus. Therefore, we suggested a glucocorticoids‐nNOS‐NO‐ONOO•‐5‐HT_1A_ receptor pathway, in the ventral hippocampal DG, implicated in the pathology of stress‐related anxiety.

## CONFLICT OF INTEREST

The authors declare no conflict of interest.
